# Adult neurogenesis does not explain the extensive post-eclosion growth of *Heliconius* mushroom bodies

**DOI:** 10.1098/rsos.230755

**Published:** 2023-10-25

**Authors:** Amaia Alcalde Anton, Fletcher J. Young, Lina Melo-Flórez, Antoine Couto, Stephen Cross, W. Owen McMillan, Stephen H. Montgomery

**Affiliations:** ^1^ School of Biological Sciences, University of Bristol, Bristol BS8 1TQ, UK; ^2^ Department of Zoology, University of Cambridge, Cambridge CB2 3EJ, UK; ^3^ Smithsonian Tropical Research Institute, Gamboa, Panama; ^4^ Wolfson Bioimaging Centre, University of Bristol, Bristol, UK

**Keywords:** Kenyon cells, neurodevelopment, proliferation, plasticity

## Abstract

Among butterflies, *Heliconius* have a unique behavioural profile, being the sole genus to actively feed on pollen. *Heliconius* learn the location of pollen resources, and have enhanced visual memories and expanded mushroom bodies, an insect learning and memory centre, relative to related genera. These structures also show extensive post-eclosion growth and developmental sensitivity to environmental conditions. However, whether this reflects plasticity in neurite growth, or an extension of neurogenesis into the adult stage, is unknown. Adult neurogenesis has been described in some Lepidoptera, and could provide one route to the increased neuron number observed in *Heliconius.* Here, we compare volumetric changes in the mushroom bodies of freshly eclosed and aged *Heliconius erato* and *Dryas iulia*, and estimate the number of intrinsic mushroom body neurons using a new and validated automated method to count nuclei. Despite extensive volumetric variation associated with age, our data show that neuron number is remarkably constant in both species, suggesting a lack of adult neurogenesis in the mushroom bodies. We support this conclusion with assays of mitotic cells, which reveal very low levels of post-eclosion cell division. Our analyses provide an insight into the evolution of neural plasticity, and can serve as a basis for continued exploration of the potential mechanisms behind brain development and maturation.

## 1. Introduction

Neural plasticity refers to the nervous system's capacity to change physically and functionally [1], and is a common feature among both vertebrates and invertebrates [2,3]. Typically, these modifications can be triggered by experience [4–7], age [8,9] or injury [10,11]. Neural plasticity has been well documented in many different arthropods [8,12,13], particularly in the mushroom body, which plays a key role in sensory integration, learning and memory [14]. This neuropil is comprised of parallel fibres formed by intrinsic neurons called Kenyon cells, which are located in the dorsal protocerebrum [15]. In the calyx, Kenyon cells make synaptic connections with projection neurons carrying sensory information from peripheral neuropils, and then project axons anteriorly through the pedunculus, to form the lobes [15]. Mushroom body plasticity can be driven by both age, occurring regardless of extrinsic factors [7,16,17], and experiential and environmental factors. As might be expected of a structure involved in learning and memory, environmentally induced plasticity in mushroom body volume has been observed in many insect species [8,16,18].

While mushroom body plasticity appears relatively common across species, the cellular mechanisms that underpin age- and experience-related volumetric expansion appear to vary [16,19,20]. One mechanism to produce neural plasticity is the continuation of neuron proliferation into adulthood (for review, see [21]) and adult production of the intrinsic mushroom body neurons, the Kenyon cells, has been described in a phylogenetically broad sample of insects, including Orthoptera, Coleoptera and Lepidoptera [22–25]. This process can be environmentally sensitive [26,27] and can play a role in learning and memory [28]. However, neural proliferation is not a requirement for mushroom body plasticity. For example, Hymenoptera, among the most advanced learners of any insects [29,30], lack adult neurogenesis [17,31] and volumetric expansion of the mushroom body is instead explained by the growth of neural processes, rather than the addition of new neurons [16]. Hence, two mechanisms—neural proliferation and neural growth—could explain the widespread evidence of volumetric plasticity in mushroom body size. However, the relative contributions of these effects, and their influence on behaviour in different species, are unclear, and our current understanding of these mechanisms is limited due to sparse sampling of insect species.

Regardless, mushroom body plasticity likely supports the acquisition or refinement of learnt behaviours. As such, closely related taxa which vary in their reliance on learning and memory may provide useful systems for exploring the function, development and evolution of neural plasticity. In this context, Heliconiini butterflies present an interesting case study in neural specialization. Within this Neotropical tribe, *Heliconius* have mushroom bodies that are up to four times larger than other Heliconiini genera, relative to overall brain size [32–34], and the largest described in any lepidoptera [34,35]. In addition, both age- and environment-dependent plasticity are linked to volumetric increases in the mushroom bodies [33], which show a consistently high degree of plasticity across *Heliconius* compared to other brain regions [36]. The likely behavioural relevance of this mushroom body expansion is a unique dietary innovation, whereby adult *Heliconius* collect and digest pollen from a restricted range of floral plants [37,38]. *Heliconius* are thought to display allocentric trap lining behaviour, based on learnt locations of preferred pollen resources [39]. This spatially and temporally faithful foraging strategy requires enhanced spatial learning and long-term memory [34], which is likely facilitated by plasticity in the mushroom bodies. However, a degree of volumetric plasticity is also reported in *Agraulis vanillae*, a non-pollen feeding member of the Heliconiini tribe [40]. In this study, we provide the first anatomical and quantitative assessment of (i) whether the mushroom bodies of a pollen feeding *Heliconius* display more age-related plasticity than a related, non-pollen feeding Heliconiini butterfly, *Dryas iulia*; and (ii) whether this plasticity is associated with increased numbers of intrinsic neurons, and the presence of adult neurogenesis.

## 2. Material and methods

### 2.1. Animal husbandry

We studied two Heliconiini species, *Heliconius erato* and *Dryas iulia*. To assess age-dependent plasticity we sampled freshly eclosed and mature individuals, aged by 17 days in standardized conditions. Individuals were reared from stocks established with locally caught, wild butterflies using the insectaries at the Smithsonian Tropical Research Institute in Gamboa, Panama. Stock butterflies were kept in 2 × 2 × 3 m mesh cages in ambient conditions. Larvae were reared in mesh pop-up cages on *Passiflora biflora*. For subsequent developmental experiments, additional stock populations were established at the School of Biological Sciences, University of Bristol, from pupae supplied by commercial breeders. Here, butterflies were maintained at 24°C–40°C and 80% humidity in approximately 2 m^3^ mesh cages in a greenhouse facility. Larvae were reared on *Passiflora biflora* and *Passiflora triloba*. In both contexts, butterflies were fed every other day with a sugar solution with an amino acid supplement (5% Vetark Critical Care Formula, 20% sugar, 75% water). For the butterfly stocks, fresh flowers were also provided from *Lantana* and *Psiguria* as additional pollen sources.

### 2.2. Immunohistochemistry (IHC)

For butterflies reared in Panama, the heads were removed in HEPES-buffered saline (HBS; 150 mM NaCl; 5 mM KCl; 5 mM CaCl_2_; 25 mM sucrose; 10 mM HEPES; pH 7.4), fixed for 16–20 h at room temperature in zinc-formaldehyde ZnFA (0.25%, 18.4 mM ZnCl2; 0.788%, 135 mM NaCl; 1.2%, 35 mM sucrose; 1% formaldehyde), before the brain was dissected in HBS [41]. After 2 h incubation in Dent's solution in 80% methanol, 20% dimethyl sulfoxide (DMSO), brains were stored in 100% methanol at −20°C. Brains were rehydrated in methanol series (90%, 70%, 50%, 30%, 0% in 0.1 M Tris buffer, pH 7.4) for 10 min each solution, and then cut into 80*μ*m sections using a Leica VT1000 S vibrating blade microtome. These sections were permeabilized for 2 h in PBSd–NGS (NGS; 5% Normal Goat Serum; DMSO; 1% dimethyl sulfoxide; 0.005% NaN3 in 0.1 M PBS), and stained with a synaptic marker, Anti-SYNORF1 (3C11, obtained from the Developmental Studies Hybridoma Bank (DSHB), University of Iowa, Iowa City, IA (RRID: AB_2315424) at 1 : 30 dilution, and a neural marker, horseradish peroxidase (HRP, Sigma-Aldrich P7899) at 1 : 5000 dilution, targeting neuron membranes, for 3.5 days at 4°C. The Panamanian samples were then washed three times in 0.1 M phosphate buffered saline (PBS) for 2 h, and counterstained with Cy2-conjugated anti-mouse at 1 : 100 and Cy3-conjugating anti-rabbit (both *Jackson ImmunoResearch Laboratories*, *West Grove*, PA) at 1 : 200 for 2.5 days at 4°C. These samples were washed again in PBS (3 × 2 h), prior to staining with DAPI (targeting cell nuclei; Sigma-Aldrich D9542) at 1 : 1000 in 0.2% Triton and H2O for 30 min at room temperature. Slices were washed in 0.2% Triton in H_2_O, and four times in 0.2% Triton in PBS for 10 min each. They were then submerged in 60% glycerol in PBS overnight, before being mounted in 80% glycerol. For butterflies reared in Bristol, a similar fixation and dissection procedure was followed but without tissue sectioning. These whole brains were stained with phospho-histone h3 (pH3; *Sigma-Aldrich*, #H0412), which labels mitotic cells [42–44], for 3.5 days at 4°C, before washing and staining with Hoechst 3342 (*Thermo-Fisher*, #H3570) in 0.1 M PBS for 3 h. Brains were then washed in PBS (3 × 30 min), clarified in a glycerol series in 0.1 M Tris (1% DMSO in 0.1 M Tris): 1%, 2%, 4% for 2 h each, 8%, 15%, 30%, 50%, 60%, 70% and 80% for 1 h each. 100% ethanol was added for 30 min and repeated three times. Finally, for clarification, methyl salicylate was carefully added into beneath the ethanol, the brains were allowed to sink, and the ethanol layer was subsequently removed, before repeating with fresh methylsalycylate. We stained brains of early pupae (P, approximately 12 h after pupation) as a positive control where pH3 labelling is expected, and newly eclosed (day 0; A0) and mature (day 7; A7) adult brains of *Dryas iulia* (P: *n* = 5, A0: *n* = 4, A7: *n* = 4) and *Heliconius erato* (P: *n* = 4, A0: *n* = 4, A7: *n* = 5).

### 2.3. EdU proliferation assay

EdU labelling was performed according to the protocol developed by Alcalde *et al*. [45] using the Proliferation Kit for Imaging, Alexa Fluor 488 dye (*Thermo-Fisher*, #C10420). Briefly, adults were first anesthetized by leaving them for 2 min at −20°C. A small window was opened in the head cuticle of young pupa and adult butterflies. They were then incubated in 20 µM EdU solution diluted in Grace's Medium (*Thermo-Fisher*, #11595030) for 3 h. After the incubation brains were dissected out and fixed in 4% paraformaldehyde (PFA) in 0.1 M phosphate-buffered saline (PBS; 7.4 pH), in 4% PFA for 10–14 h. Brains were washed using 0.1%Triton in 0.1 M PBS (PBS-T) and permeabilized in 1%Triton in 0.1 M PBS. The click-it reaction was performed as instructed by the manufacturer (*Thermo-Fisher*, #C10420), and 500 µl of the reaction mix was added per well, each of which contained three brains. The brains were incubated in the reaction mix for 30 min, protected from light. Brains were washed in 0.1% PBS-T twice and 0.1 M PBS for the third wash (30 min each). Nuclei were marked using Hoechst 3342 in 0.1 M PBS for 3 h. Brains were then washed 3 times in 0.1% PBS-T. Finally, they were clarified as described above. We stained brains of early pupae as control where neurogenesis is expected, and brains from newly eclosed (day 0; A0) and matured adults (day 7; A7) of *Dryas iulia* (P: *n* = 4, A0: *n* = 2, A7: *n* = 1) and *Heliconius erato* (P: *n* = 4, A0: *n* = 2, A7: *n* = 2).

### 2.4. Microscopy and image acquisition

Samples were imaged using a Leica TCS SP5 confocal laser-scanning microscope. For the cell counting experiment using sectioned tissue, the mushroom body calyces and the surrounding cluster of Kenyon cells were scanned with 10X dry objective (z-step 3 µm). Subsamples of the Kenyon cell cluster were then scanned at higher magnification with a 63X glycerol immersion objective with a 1.3 NA (63X HCX PL APO CS, Leica microsystems No. 11506194), and UV excitation at 405 nm wavelength. Image stacks measuring 25 × 25 × 15 µm were produced for five randomly selected areas, with a 1 µm *z*-step. Each box consisted of a z-stack of 15 images. These images were used to estimate the average density of Kenyon cells nuclei within the measured volume, which was then extrapolated across the whole volume of the Kenyon cell cluster to estimate total Kenyon cell number. For whole brains, samples were scanned using 10X and 20X dry objectives. The resolution of both sets of images was 1024 × 1024 pixels. Images obtained with the confocal microscope were edited using Fiji [46] to adjust brightness, contrast and colours using the colour and channel tools.

### 2.5. Volumetric reconstruction and cell counting

**Figure 1.**
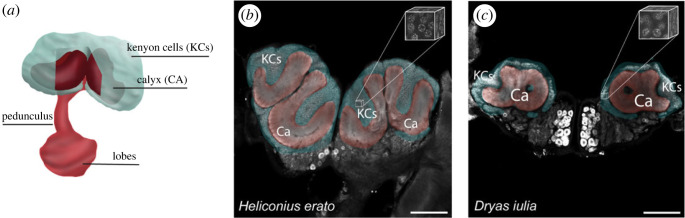
Schematic drawing of the mushroom body (*a*) and segmentations of the Kenyon cell cluster (KCs, blue) and Calyx (CA, red) of *Heliconius erato* (*b*) and *Dryas iulia* (*c*). The small boxes of 25 µm × 25 µm × 15 µm represent an example of the sections counted. Scale bars: 200 µm.

As in previous studies [33,47–49], the volume of the calyx and Kenyon cell cluster were reconstructed using the segmentation editor of Amira-Avizo 3D 2021. Anti-SYNORF1 has been used in many invertebrate species to identify neuropil boundaries [33,47–49], which enables the identification and reconstruction of different regions of the mushroom body including the Kenyon cell cluster and the calyx. After segmenting, the statistics tool was used to obtain the volumes of each structure. Cell counting was based on DAPI staining, with anti-HRP used to confirm neuronal identity. The number of Kenyon cells within each sub-sampled image stack (see above) was counted using two different approaches: manual counting as described in Couto *et al*. [34], and automatic counting. For manual counting, the anterior, medial and posterior parts of the cell nucleus (relative to the body axis) of each in the sample boxes were manually segmented and interpolated in the z-axis to the rest of the cell nucleus. The counting was blind for different age groups but not for species as the morphological differences between them are clearly identifiable. Half-cell nuclei within the limits of the box were counted as half. Automated counting was performed using Fiji [46]. We used two main plugins to calculate the number of cell nuclei within the boxes, *StarDist* and Modular Image Analysis (*MIA*). *StarDist* is a plugin that detects objects with star-convex shape priors [50]. MIA can be used to automate running *StarDist* and perform subsequent analyses on the detected objects [51]. This was revised manually to avoid error. The script for this analysis is provided in the supplementary material, with additional descriptive information in the electronic supplementary material. The automatic counting was fully blinded, and both automated and manual counts were measured for each of the five image stacks for all individuals, allowing a direct comparison between the measurements. In both cases, the average density of cells within the five sampled boxes was extrapolated to the volume of the Kenyon cell cluster for each individual (figure 1).

### 2.6. Statistical analyses

All statistical analyses were performed with R v. 3.0.2 [52]. The similarity of the manual and automated counting methods was assessed by regressing automated and manual counts, separately for each species, and analysing differences in regression parameters using *SMATR* [53]. In addition, a formal test of agreement between methods was performed following Salinas *et al*. [54], using the Tukey Mean-Difference method [55,56]. These analyses revealed a high degree of consistency, suggesting the automated counts can provide a rapid assessment tool for future studies (see electronic supplementary material, Information). For our analyses we therefore used the automatic counts. To compare the number of Kenyon cells and volume of the neural structure volumes, we used generalized linear mix models GLMM in the *glmmTMB* package v 1.1.2.3 [57]. In the models, age and species were the fixed factors. Sex was initially included as a random factor, but did not improve model fit and was therefore removed from the final models. The significance of the models was calculated using ANOVAs with the *car* package v. 3.0-12 [58]. Pairwise comparisons were performed with the emmeans package v1.7.0 [59] and they were corrected using Tukey's test.

## 3. Results

### 3.1. Post-ecolosion volumetric expansion of *Heliconius* mushroom bodies does not occur through net addition of new Kenyon cells

**Figure 2.**
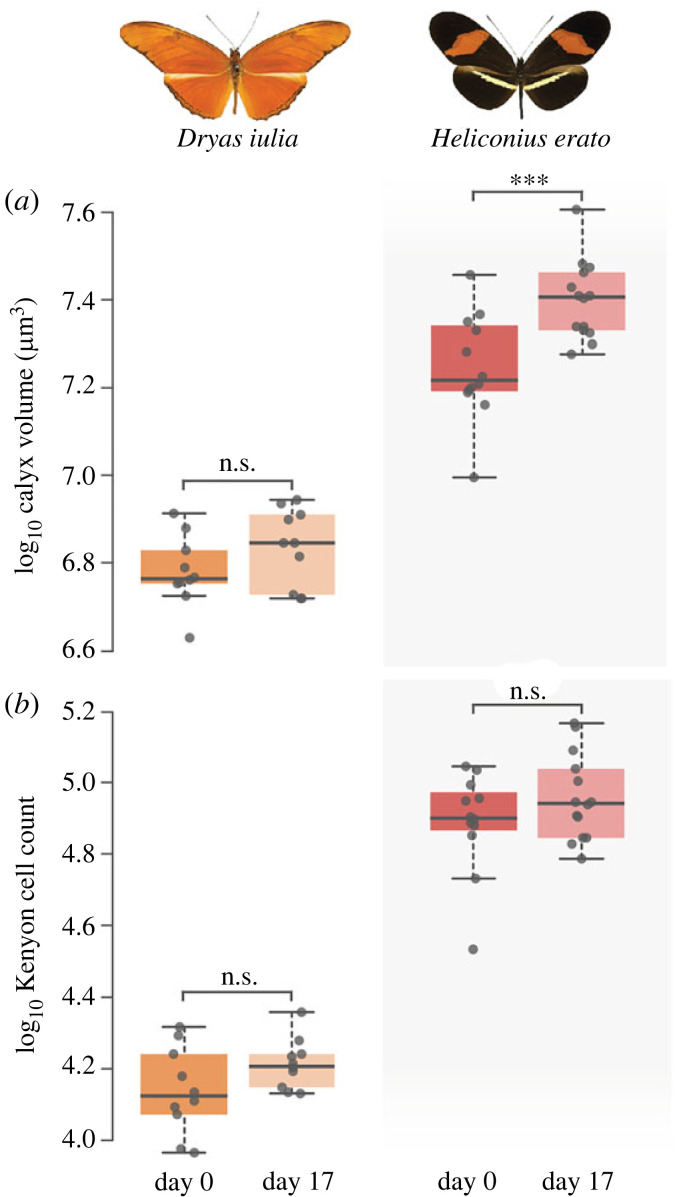
Age dependent variation in the volume of the mushroom body calyx (*a*) but not in Kenyon cell number (*b*). Boxes encompass the two middle quartiles with central line showing the median. Whiskers extend to furthest data point within 1.5 times the interquartile range. ***p < 0.001.

As expected, for age matched samples, *Heliconius erato* showed significantly higher values than *Dryas iulia* for calyx volume and Kenyon cell number (figure 2, Tables S3/S4). In *Dryas iulia*, old and young individuals did not significantly differ in calyx volume (*t*_42_ = −1.305, *p* = 0.565) (figure 2, electronic supplementary material, Table S4). By contrast, older *Heliconius erato* exhibited a significant increase in calyx volume (*t*_42_ = −4.219, *p* ≤ 0.001). Older individuals of both species had higher Kenyon cell densities (*Dryas iulia*: *t*_44_ = −5.652, *p* < 0.001; *Heliconius erato*: *t*_44_ = −5.534, *p* < 0.001; electronic supplementary material, figure S3, and table S4). However, this may be explained by a tendency for the volume of the Kenyon cell cluster to decrease with age (*Dryas iulia*: *t*_42_ = 2.439, *p* = 0.085; *Heliconius erato*: *t*_42_ = 2.023, *p* = 0.196; electronic supplementary material, figure S3, and table S4). Neither *Dryas iulia* (*t*_41_ = −1.481, *p* = 0.457) nor *Heliconius erato* (*t*_41_ = −1.567, *p* = 0.408) showed evidence of increased numbers of Kenyon cells with age (figure 2, electronic supplementary material, table S4).

### 3.2. Labelling of mitotic cells confirms the absence of extensive adult neurogenesis in the mushroom bodies

**Figure 3.**
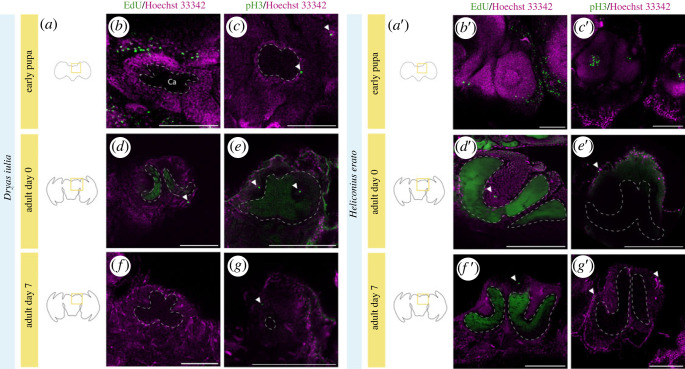
Little evidence of adult neurogenesis in Heliconiini mushroom bodies. (*a,a′*), Schematic drawings of the brain of butterflies. Nuclei and EdU staining in *Dryas iulia* (*b,d,f*) and *Heliconius erato* (*b′,d′,f′*). Nuclei and pH3 staining in *Dryas iulia* (*c,e,g*) and *Heliconius erato* (*c′,e′,g′*). Scale bars = in (*b,b′,c*) and (*c′*); 200 µm in (*d–f*) and (*d′–f′*). White arrows indicate EdU + or pH3 + cells. Scale bars = 100 µm in (*b,b′,c*) and (*c′*); 200 µm in (*d–g*) and (*d′–g′*).

**Figure 4.**
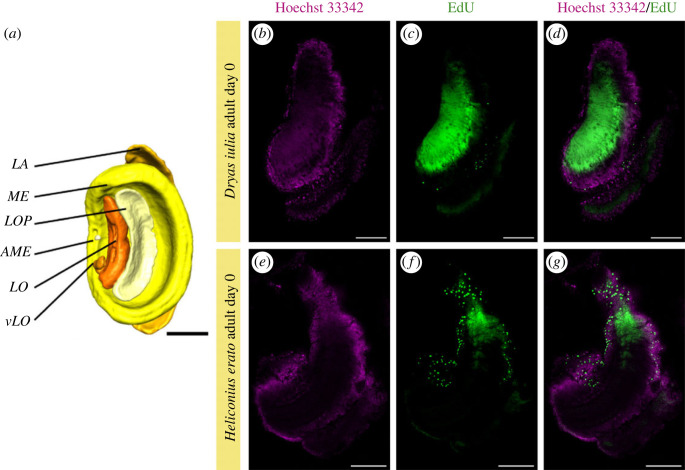
Adult neurogenesis in the optic lobe (OL) of 0 days adult Heliconiini butterflies. (*a*). Parts of the optic lobe from Montgomery *et al*. [33]. (*b,e*) Nuclei staining. (*c,f*) EdU staining. (*d*,*g*) Nuclei and EdU double staining. LA: lamina, ME: medulla, AME: accessory medulla, LO: lobula, LOP: lobula plate, vLO: ventral lobe of the LO. B: Scale bars = 200 µm.

Using two methods to label proliferating cells, we sought to confirm that the lack of age-dependent variation in Kenyon cell number reflects a lack of adult neurogenesis (electronic supplementary material, figure S4). In our EdU labelling experiments, only one or two individual cells showed some labelling in adult brains (in 1 out of 2 A0 Dryas, 2 out of 2 A0 and 1 out of 2 A7 *Heliconius;* figure 3). Using immunolabelling against pH3, a mitotic marker, neither species showed evidence of widespread pH3 labelling (figure 3). Only between 1 to 5 labelled nuclei were observed in adult *Dryas iulia* (in 3 out of 4 A0 and 2 out of 4 A7 brains) and *Heliconius erato* (in all A0 and A7) (figure 3). The location of nuclei labelled by pH3 in A0 *Dryas iulia* were in the middle of the cups which form the calyces of the mushroom body where neuroblasts are located in other species [15,60]. In A7 *Dryas iulia* only 1 to 2 nuclei were labelled but here they were in the cells surrounding the outer regions of mushroom body calyx, where proliferating neuroblasts are not expected. In A0 *Heliconius erato* the 2 to 4 nuclei that were labelled in some individuals were also in the cells surrounding the mushroom body (figure 3), a position which is again inconsistent with the expected location of Kenyon cell progenitor cells. Only in one individual Heliconius A0 were two EdU + cells found in the middle of the Kenyon cell cluster (figure 3). The staining in the pupal stages of both species is notably much stronger (figure 3), indicating that the level of cell division in the adult mushroom body is comparatively very low. In addition, EdU labelling revealed a considerable number of cells (30 + nuclei) marked in the optic lobe in newly eclosed individuals from both species, and more numerously (100 + nuclei) in *Heliconius* (figure 4). This more extensive labelling again in the optic lobes contrasts with the low levels observed in the mushroom bodies.

## 4. Discussion

Among butterflies, *Heliconius* have a unique foraging strategy reliant on learning the spatial distribution of floral resources. Associated with this behaviour, they also have the largest mushroom bodies of any Lepidoptera described to date [34,35]. Previous analyses have established a significant influence of age and environmental effects on mushroom body size in *Heliconius* [33,36]. Here, we contribute two advances to our understanding of this system. First, we provide evidence that mushroom body plasticity is more extensive in *Heliconius erato* than in *Dryas iulia*. Second, we combined data from Kenyon cell counts and mitotic markers to rule out a predominant role of adult neurogenesis in shaping mushroom body plasticity in Heliconiini.

Age-dependent plasticity has been described in the mushroom body of a range of insects, and is particularly well studied in Hymenoptera, including *Apis mellifera* [16,61], *Bombus impatiens* [8] and *Cataglyphis fortis* [62]. We find similar patterns of post-eclosion growth in the calyx of *Heliconius erato*, which is less pronounced in *Dryas iulia* (figure 2, electronic supplementary material, tables S3 and S4). We note our results for *Dryas iulia* differ from a previous study in another non-pollen feeding Heliconiini, *Agraulis vanillae* which reported more significant age and environmental effects on calyx volume [40]. While a direct comparison with this study is not possible due to differences in methodology and statistical analysis, this may support our interpretation of the trend detected in *Dryas* as being indicative of some plasticity [5]. However, in contrast to the age dependent effects on calyx volume, the consistency in Kenyon cell number between old and young butterflies in both species is striking. This result implies the volumetric expansion of the calyx with age is not due to the continuation of neurogenesis into adulthood (figure 2, electronic supplementary material, tables S3 and S4). We did observe slightly smaller volumes of the Kenyon cell cluster in aged individuals of both species, leading to significantly higher Kenyon cell densities. A comparable decline in Kenyon cell cluster volume was noted in bees as they aged or transitioned between different labour roles volume [63]. We suggest that this can most simply be explained by the increased volume of the calyces pushing the Kenyon cell cluster against the external membrane of the brain, reducing its volume and packaging the cell bodies more tightly.

The cell count data alone is, however, insufficient to rule out the presence of adult neurogenesis, as there may be an interaction between cell production and cell death that could result in a turnover of cells. Indeed, adult neurogenesis has been previously reported in the adult brain of *Agrostis ipsilon,* a Lepidopteran [25], and Panov [60,64] also provided evidence of persistent neuroblasts in adults of multiple species of Lepidoptera using histological images. Hence, to fully explore the possibility of adult neurogenesis, we used EdU and pH3 labelling to test for cell proliferation in the adult brains of Heliconiini butterflies. Our results using pH3 labelling showed a very low degree of proliferation in the mushroom bodies of adult Heliconiini butterflies (figure 3). In *Dryas iulia* 2–4 labelled nuclei were found within the cups of the calyces, suggesting they were cells in mitosis (figure 3), but by day 7 there was no sign of cell division in the centre of the calyx, and only 2–3 labelled cells surrounding the Kenyon cell cluster (figure 3). Both EdU and pH3 mark all cells in proliferation so we cannot confirm if our results show neurogenesis, as opposed to gliagenesis or oligogenesis, for example. However, in other insects with adult neurogenesis, new-born neurons come from persistent neuroblasts located in the cell cluster surrounding the calyx [20,24,25]. The location of the labelled nuclei within the cup of the *Dryas* mushroom body calyx is therefore potentially consistent with them being neural progenitor cells.

In *Heliconius*, butterflies also presented pH3 positive and EdU positive nuclei only at very low numbers, typically approximately 3–5 nuclei per hemisphere (figure 3). However, in *Heliconius* the marked nuclei were mainly located on the outer edge of the Kenyon cell cluster, which we interpret as being inconsistent with the predicted locality of mushroom body neuroblasts and newly born Kenyon cells. Adult neurogenesis has been reported in a range of other insects, including *Tribolium castaneum* [24], *Achaeta domesticus* [22], *Agrostis ipsilon* [25], and a few Coleoptera [20]. In Coleoptera, 17 h after the injection with BrdU (a similar labelling technique to Edu) *Zophobas* sp. showed 14 cells marked on the calyces of the mushroom bodies, *Tenebrio molitor* 7 cells, and *Harmonia axyridis* 3 [20]. In *Agrostis ipsilon*, 2 days after BrdU injection 8–12 cells were marked in the mushroom body [25]. The EdU incubation in adults in our protocol lasted only three hours, so it is possible that some variation in number is caused by longer incubation times in these previous studies. Regardless, the levels of mitotically active cells that we detected in the adult mushroom bodies of *Heliconius erato* were very low, and possibly consistent with levels seen in other species.

Our data suggest Heliconiini therefore exhibit age and environmentally determined neural plasticity in a manner similar to Hymenoptera. In the mushroom bodies of *Achaeta domestica* [19,26,28] and *Tribolium castaneum* [24,27] neuron proliferation mediates olfactory based neural plasticity. Likewise, in *Agrostis ipsilon* neural proliferation in the adult mushroom body has been hypothesized to be linked to odour dependent plasticity [25]. By contrast, in Hymenoptera, the branching of Kenyon cell dendrites is the main factor associated with mushroom body plasticity, rather than continued production of Kenyon cells [16]. This scenario is more consistent with our data as, regardless of whether the few labelled nuclei we observed are the product of adult neurogenesis, they appear to be insufficient to explain the post-eclosion mushroom body growth we observed in *Heliconius erato*, or the differences observed between the species.

Interestingly, we did observe clear signs of cell proliferation in the optic lobes of newly eclosed adults of *Heliconius* and *Dryas* butterflies (figure 4). Neural precursors in the optic lobes of adults have also been reported in *Agrostis ipsilon* [25], *Achaeta domesticus* [22], *Drosophila melanogaster* (albeit at very low levels) [65], and in other insects [66,67]. The role of adult neurogenesis in the optic lobes is currently unknown, however *Heliconius erato* have been shown to have age-dependent volumetric plasticity in the optic lobes [33]. As Heliconiini butterflies use visual cues to navigate between resources [39,68] and discriminate hostplants [69], floral cues [70–72] and mating cues [73–77], we suggest the continued development of the optic lobes into adulthood may have important behavioural effects. Of potential relevance is the observation that *Heliconius* are not behaviourally mature until approximately 8 days post eclosion [78].

In summary, we found considerable post-eclosion expansion of the mushroom bodies in *Heliconius erato*, while the closely related Heliconiini *Dryas iulia* showed no such growth, suggesting a pronounced difference in neuroplasticity between the two species. Using cell count data, and two independent methods of quantifying mitotic activity in developing brains, we further show that adult neurogenesis is absent or occurs at very low levels in the mushroom bodies in both of these species. These data imply that the post-eclosion volumetric expansion of mushroom bodies seen in *Heliconius erato* is unlikely to be a result of neurogenesis, but rather the growth of existing neurons, similar to what is known in Hymenoptera [16]. This provides a foundation for further exploring the role of neural plasticity, particularly synaptic reorganization, in the cognitive differences between *Heliconius* and other Heliconiini genera.

## Data Availability

Data on the cell counts and the nuclei counting script are included in the electronic supplementary material information [79]. Data files are also deposited on Dryad Digital Repository: https://datadryad.org/stash/share/FL9EVkGYYN_6rVkxHCxCC4MCCjJ0HzQn2WVusLdAl9A [80].
